# Influence of the Rearing Host on Biological Parameters of *Trichopria drosophilae*, a Potential Biological Control Agent of *Drosophila suzukii*

**DOI:** 10.3390/insects10060183

**Published:** 2019-06-25

**Authors:** Svetlana Boycheva Woltering, Jörg Romeis, Jana Collatz

**Affiliations:** 1 Research Division Agroecology and Environment, Agroscope, Reckenholzstrasse 191, 8046 Zurich, Switzerland; svetlana.boycheva-woltering@uni-konstanz.de (S.B.W.); joerg.romeis@agroscope.admin.ch (J.R.); 2 Department of Biology, University of Konstanz, Universitätsstrasse 10, 78464 Konstanz, Germany

**Keywords:** local adaptation, host choice, phenotypic plasticity, biocontrol

## Abstract

*Trichopria drosophilae* is a pupal parasitoid that can develop in a large number of drosophilid host species including the invasive pest *Drosophila suzukii*, and is considered a biological control agent. We investigated the influence of the rearing host on the preference and performance of the parasitoid, using two different strains of *T. drosophilae*, reared on *D. melanogaster* or *D. suzukii* for approximately 30 generations. Host switching was employed to assess the impact of host adaptation on *T. drosophilae* performance. In a no-choice experimental setup, *T. drosophilae* produced more and larger offspring on the *D. suzukii* host. When given a choice, *T. drosophilae* showed a preference towards *D. suzukii*, and an increased female ratio on this host compared to *D. melanogaster* and *D. immigrans*. The preference was independent from the rearing host and was confirmed in behavioral assays. However, the preference towards *D. suzukii* increased further after a host switch from *D. melanogaster* to *D. suzukii* in just one generation. Our data indicate that rearing *T. drosophilae* for several years on *D. melanogaster* does not compromise its performance on *D. suzukii* in the laboratory. However, producing a final generation on *D. suzukii* prior to release could increase its efficacy towards the pest.

## 1. Introduction

Parasitoid immatures develop in or on a single arthropod host, whereas the adults are free living. Thus, their development takes place in an intimate relationship with their host, and the number of suitable host species is often limited. The host range of parasitoid species is determined, on one hand, by the nutritional suitability of the host and the ability of the parasitoid to overcome host defenses (physiological host range), and on the other hand, by environmental circumstances such as the habitat use of hosts and biological interactions with competitors, pathogens, and host plants (realized host range) [1]. Within a species, parasitoids may also form host-associated populations that gradually adapt to the use of a particular host [2]. Early steps in the formation of these populations may be the learning of host-associated cues during early life stages [3,4], and assortative mating within the population [2], whereas genetic adaptation to the host species takes place over several generations [5]. Consequently, host switches may come at a fitness cost, since the parasitoid needs to adapt to the host physiology and its defense system [5]. 

The host range and potential of parasitoids to adapt to new hosts are important elements to be considered in biological control programs [6,7,8]. During mass rearing prior to release, parasitoids may adapt to the rearing host as a form of artificial evolution or local adaptation [7,9]. In particular, if rearing is conducted on a species different from the target host, this may have consequences for the risk to non-target species and the efficacy on the target. However, mass-rearing on alternative hosts may be necessary to avoid the risk of spreading pests along with the natural enemy [10], or due to the fact that the target host is difficult and/or expensive to rear [11,12]. Under such circumstances, it is crucial to assess the influence of the alternative rearing host on biological parameters of the parasitoid to optimize the biological control program.

Biological control with parasitoid wasps is currently being discussed for the spotted wing Drosophila, *Drosophila suzukii* (Matsumura; Diptera: Drosophilidae), an invasive species native to Asia. It has been introduced into Europe and the Americas in the past decade, and is now firmly established in large parts of these continents [13,14]. Unlike most other *Drosophila* species, *D. suzukii* possesses a serrated ovipositor, which allows it to lay its eggs in ripening undamaged berries, stone fruits, and grapes. Economic damages caused by *D. suzukii* in these crops is high [15,16], and has spurred research into sustainable control options [17,18], such as the augmentation of native natural enemies [19,20,21,22]. 

In particular, parasitoid wasps, and among those, the pupal parasitoid *Trichopria drosophilae* (Perkins; Hymenoptera: Diapriidae), have received much attention as potential biological control agents for *D. suzukii* [23,24,25,26,27]. The species has been recorded in Asia (Korea: [28] China: [27]), America (California: [23], Mexico: [29]), and Europe (Spain: [19], Italy: [30], Switzerland [31], France [32]). Parasitization rates of *D. suzukii* by *T. drosophila* are high under laboratory conditions [32,33], and rearing in large numbers is feasible. The solitary parasitoid emerges with a high number of mature eggs [23], and can parasitize more than 64 hosts during its lifetime [27]. First experiments for field releases in Europe have been undertaken, with encouraging results in some cases [26,34].

Although individuals that emerge from *D. suzukii* hosts are larger [23] and presumably have a higher egg load than those from *D. melanogaster* [26], commercial rearing of *T. drosophilae* currently takes place with *Drosophila melanogaster* Meigen (Diptera: Drosophilidae) as the host [26]. This species is less demanding in terms of food quality and rearing conditions, has a faster generation cycle compared to *D. suzukii*, and thus can be easier to rear in large quantities ([35]; own observations). However, while other common parasitoids of *Drosophila* pupae, such as *Pachycrepoideus vindemmiae* (Rondani) and *Spalangia erythromera* Förster (both Hymenoptera: Pteromalidae) are ectoparasitoids, *T. drosophilae* is an endoparasitoid [23]. It presumably lives in a closer association with its host, and thus could be particularly affected by the species it is reared on.

Therefore, we wanted to test if the rearing host (*D. melanogaster* or *D. suzukii*) influences efficacy of *T. drosophilae* as biocontrol agent of *D. suzukii*. In particular, we wanted to know (i) if the host species influences performance and host preference of offspring, and (ii) if local adaptation to the rearing host takes place under the rearing conditions. As an independent reference, we included *Drosophila immigrans* as a third host species, with which the two parasitoid strains had no previous encounter. 

## 2. Materials and Methods 

### 2.1. Insects

Cultures of *D. suzukii* and *Drosophila immigrans* Sturtevant (Diptera: Drosophilidae) were established from about 100 and 25 individuals, respectively, collected in Zurich, Switzerland in 2013. The culture of *D. melanogaster* originated from a wild-type strain from Professor Walter J. Gehring’s Lab (University of Basel, Switzerland). All fly larvae were reared on an artificial diet (400 g Banana, 20 g agar-agar, 50 g brewer’s yeast, 30 g wheat flour, 20 g saccharose, 4 g nipagin, 1 L water). Adult flies were kept in flight cages (32 × 22 × 16 cm) and could directly oviposit onto the diet (*D. melanogaster*, *D. immigrans*), or were kept in plastic jars (11 cm dia., 15 cm height) with a fine metal grid at the bottom, which were placed onto the artificial diet and through which oviposition could take place (*D. suzukii*). The diet was replaced three times per week, and adult flies were replaced every four weeks. 

About 50 individuals of *T. drosophilae* (TD) were collected in Mezzana Ticino (Switzerland) in 2014 [31] and were reared for three generations on *D. melanogaster* (DM). Half of the parasitoids were then transferred to *D. suzukii* (DS) as host, and both lines (TD-DM and TD-DS) were subsequently reared separately on their respective hosts for more than 30 generations (2.5 years). 

For rearing, parasitoids were kept in flight cages with access to water and honey, and were provided twice a week with Drosophila pupae that had pupated on dental cotton rolls (12 mm dia., Gerber Instruments, Effretikon, Switzerland) for parasitization. For a detailed description of parasitoid and fly rearing, see Knoll et al. [31].

For the experiments, paper towels instead of cotton rolls were used as a pupation substrate for the flies. This allowed easier separation of the pupae by cutting the paper. The paper was placed into rearing pots of all three *Drosophila* species and left overnight. Papers carrying fly pupae were removed the next morning and stored for another 24 h before use in the assays.

All rearing and experiments were conducted in climate chambers at 22 °C, 70% RH and a 16:8 L:D photoperiod.

### 2.2. No-Choice Assay

To assess the suitability of the three Drosophila species as hosts for *T. drosophilae* and to detect potential genetic adaptations to a particular host species during the 2.5-year laboratory rearing, no-choice assays were conducted. Two- to five-day old mated females of the two *T. drosophilae* lines TD-DM and TD-DS were used in the experiment. At this age, females had sufficient time for mating and had a high number of mature eggs [23]. 

Each female was provided with 40 pupae of either *D. melanogaster*, *D. suzukii*, or *D. immigrans* within a rearing tube (4.5 cm dia. 4 cm height) containing a drop of honey and a wet cotton pad. After 24 h, the parasitoid was removed, the pupae were checked daily, and emerged flies and parasitoids were removed and counted. Emerged parasitoids were sexed, and females were immediately stored in 70% ethanol for biometric measurements. A total of 25 tubes was set up for each parasitoid strain with each fly species in five different runs. Likewise, a total of 25 control tubes that contained 40 pupae of one of the respective fly species were set up in parallel to assess the quality of the hosts. 

Weight was chosen as a proxy to measure parasitoid fitness, as it is typically linked to size, life-span, and fecundity [36,37,38,39]. Fifty female parasitoids per treatment were removed from the ethanol. No more than five individuals from the same mother were used. Furthermore, fifty pupae of each *Drosophila* species were collected as described above and gently cleaned in tap water from adhering diet. Pupae and parasitoids were dried for 2 h at 40 °C, and subsequently kept at room temperature for another 24 h to reach an equilibrium with outside humidity. Subsequently, pupae and parasitoids were weighed individually using an electronic balance (Mettler Toledo MX5 microbalance; ± 0.002 mg, Mettler Toledo, Columbus, OH, USA). 

### 2.3. Choice Assay

To compare parasitization and sex ratio decisions of females from the two *T. drosophilae* strains (TD-DM and TD-DS) on the three host species, choice assays were conducted. The experiments were performed as described above, except that parasitoid females were provided for 24 h with 120 pupae of the three Drosophila species at the same time (40 pupae of each species) in a larger plastic tube (10 cm dia., 15 cm height). The pupae were provided in groups of 2–8 individuals on small pieces of the paper they had pupated on, and the pieces were mixed well. After exposure to the parasitoid female, the pupae were separated by Drosophila species and kept in rearing tubes. Emerging flies and parasitoids were counted and removed. Parasitoids were sexed. A total of 25 replications were conducted for each of the two parasitoid strains. 

### 2.4. Behavioral Assay on First Host Choice

To differentiate between the effects of genetic adaptation and phenotypic plasticity on host choice, a behavioral assay was conducted. Offspring from the two parasitoid lines TD-DM and TD-DS, generated on either *D. melanogaster* or *D. suzukii*, were used in the assay, resulting in four experimental groups: TD-DM-DM, TD-DM-DS, TD-DS-DM, and TD-DS-DS. Newly emerged females from these four groups were kept with males from the same group for four to six days to ensure mating, and were then used for the experiments. At the time of the experiments, females did not have any oviposition experience. Females were individually placed in Eppendorf tubes (1.5 mL) and left to calm after handling for 10–15 min prior to the assay.

For each assay, six pupae of each *D. melanogaster* and *D. suzukii* were offered in a glass Petri dish (9 cm dia.). The pupae were provided on four pieces (2 per species; approx. 1.5 × 1.5 cm) of the paper towel on which they had pupated. The female wasps were released into the center of the Petri dish and observed for a maximum of 600 s. The first oviposition choice was noted. The wasp had made a choice when it drilled with its ovipositor into one of the pupae. As a full oviposition sequence takes 309 ± 29 s (Mean ± SE, n = 10) in *T. drosophilae*, the observation was ended once an oviposition had been recorded. Wasps that did not make a choice within the 600 s observation time were discarded. Twenty five oviposition choices were recorded for each of the four experimental groups. Pupae were exchanged after each observation, and each wasp was only used once. No more than five wasps per experimental group were tested on each day, and no more than four wasps from the same batch were used.

### 2.5. Statistical Analysis

Weight and number of emerging flies from pupae from the three host species were analyzed using non-parametric Kruskal–Wallis tests followed by Mann–Whitney U tests for multiple comparisons. Offspring numbers of *T. drosophilae* in no-choice assays were analyzed with generalized linear models (GLM), assuming Poisson error distribution and a loglink function using “parent strain” and “host species” as fixed factors, followed by a sequential Bonferroni corrected post-hoc test for the factor “host species”. Offspring numbers in choice assays were analyzed using generalized linear mixed models (GLMM) with the individual as subject and the same factors and post-hoc test as above. Comparisons between the parasitoid strains TD-DM and TD-DS on the three host species were conducted with Mann–Whitney U test. Females that had no or all male offspring were excluded from the analysis (0–7 tubes were excluded per treatment, final sample sizes n = 18–25), since those females were likely infertile or unmated, respectively. The effect of the factors “parent strain” and “host species” on the weight of female *T. drosophilae* was assessed using analysis of variance (ANOVA) on log-transformed data, followed by a Tukey post hoc test for multiple comparisons between host species. The effect of the factors “choice,” “parent strain,” and “host species” on the proportion of female offspring was analyzed with a GLM, assuming binomial error distribution and with a logit link function and robust estimator. Multiple comparisons between host species were conducted with a sequential Bonferroni corrected post-hoc test. Comparisons of the effect of choice within host pupae were performed with Mann–Whitney U tests. The effect of the factors “parent strain” and “host species” on the first host choice was analyzed with loglinear analysis. All analyses were performed with IBM-SPSS statistics version 24 (IBM Corporation, Armonk, NY, USA).

## 3. Results

### 3.1. Weight and Number of Emerging Flies from Host Pupae

Host pupae differed significantly in weight (Kruskall–Wallis ANOVA; K_2, 148_ = 99.62; *p* < 0.001), with pupae from *D. melanogaster* (1.10 mg ± 0.03; Mean ± SE) being lighter than *D. suzukii* (1.45 mg ± 0.04; U = 2091.00; *p* < 0.001) and *D. immigrans* (2.22 mg ± 0.06; U = 2495.50 *p* < 0.001), and *D. suzukii* being lighter than *D. immigrans* (U = 2246.00; *p* < 0.001). The number of emerging flies from control tubes differed between host species (Kruskall–Wallis K_2, 73_ = 24.26; *p* < 0.001), with more individuals emerging from *D. melanogaster* (37.80 ± 0.38; Mean ± SE) than from *D. suzukii* (33.32 ± 0.73; U = 76.00; *p* < 0.001) and from *D. immigrans* (33.52 ± 1.00; U = 117.00; *p* < 0.001). No difference between the latter two species was observed (U = 344.50; *p* = 0.533).

### 3.2. Offspring Number of T. drosophilae in No-Choice Assay

**Figure 1:**
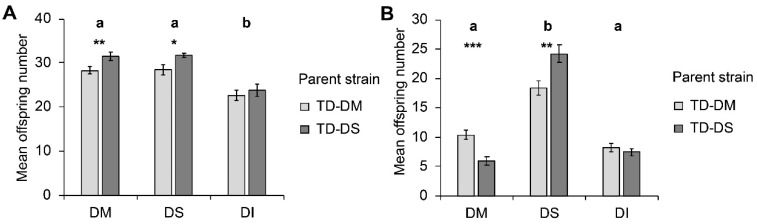
Mean (± SE) number of offspring produced by *Trichopria drosophilae* from strains reared for >30 generations on *Drosophila melanogaster* (light grey) or on *Drosophila suzukii* (dark grey) on different host species under (**A**) no-choice and (**B**) choice conditions. DM: *D. melanogaster*; DS: *D. suzukii*; DI: *D. immigrans*. Different letters above bars indicate significant differences between host species at the 5% level (generalized linear model (GLM) (**A**) and generalized linear mixed model (GLMM) (**B**), followed by sequential Bonferroni post hoc tests), stars indicate significant differences between parasitoid strains at the *p*-level of 0.001% (***), 0.01 (**), or 0.05 (*) (Mann–Whitney U test).

Host species significantly influenced parasitoid offspring number (W_2, 138_ = 47.16; *p* < 0.001), with less offspring emerging from pupae of *D. immigrans* compared to pupae of *D. suzukii* and *D. melanogaster* (Figure 1a). The parasitoid strain (TD-DM or TD-DS) also significantly influenced offspring number (W_1, 139_ = 7.53; *p* = 0.006), with individuals from the TD-DS strain having more offspring. This difference between strains was significant for *D. melanogaster* (*p* = 0.006) and for *D. suzukii* (*p* = 0.026), but not for the less suitable host *D. immigrans* (*p* = 0.140). The interaction between parasitoid strain and host species was non-significant (W_2, 138_ = 0.539; *p* = 0.764). 

### 3.3. Weight of Female T. drosophilae from No-Choice Assays

**Figure 2:**
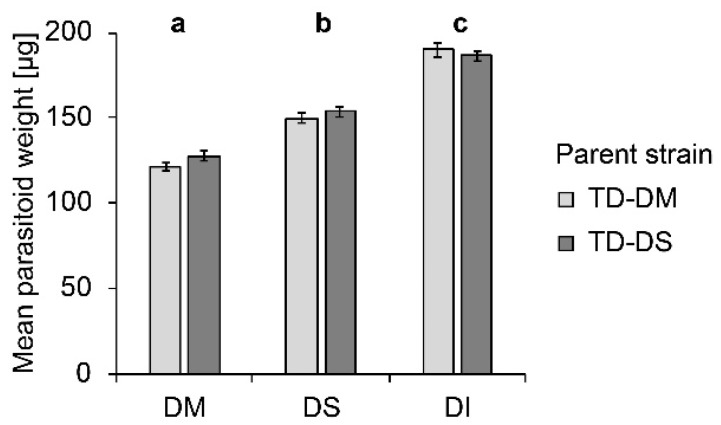
Mean (± SE) weight of female offspring produced by *T. drosophilae* from a strain reared for >30 generations on *D. melanogaster* (light grey) or on *D. suzukii* (dark grey) on different host species under no-choice conditions. DM: *D. melanogaster*; DS: *D. suzukii*; DI: *D. immigrans*. Different letters above bars indicate significant differences between host species at the 5% p-level (ANOVA followed by Tuckey post hoc tests). No significant differences between strains were detected (Mann–Whitney U test).

Host species significantly influenced the weight of female parasitoid offspring (F_2, 293_ = 233.97; *p* < 0.000). Females emerging from *D. melanogaster* pupae were lightest, those emerging from *D. suzukii,* intermediate, and from *D. immigrans,* heaviest (Figure 2). No influence of the parasitoid strain (F_1, 294_ = 1.65; *p* = 0.200) on female offspring weight was visible, and no interaction between parent strain and host species occurred (F_2, 293_ = 1.66; *p* = 0.193).

### 3.4. Offspring Number of T. drosophilae in Choice Assay

In choice assays, host species significantly influenced offspring number (F_2, 105_ = 162.055; *p* < 0.001) as well as parasitoid strain (F_1, 105_ = 4.501; *p* = 0.036) (Figure 1b). When given the choice, female *T. drosophilae* produced more offspring on *D. suzukii* than on *D. melanogaster* or *D. immigrans* within the 24 h of the experiment. Furthermore, a significant interaction between the factors parasitoid strain and host species was observed (F_2, 105_ = 18.530; *p* < 0.001). The preference for *D. suzukii* was significantly stronger in the TD-DS strain than in the TD-DM strain, i.e., on the host species *D. melanogaster*, TD-DM produced significantly more offspring than TD-DS (*p* < 0.001), whereas on the host species *D. suzukii*, it was the opposite (*p* = 0.001). No significant influence of the parent strain was visible for the number of offspring that emerged from *D. immigrans*. 

### 3.5. Proportion Females of T. drosophilae in Choice and No-Choice Assay

**Figure 3:**
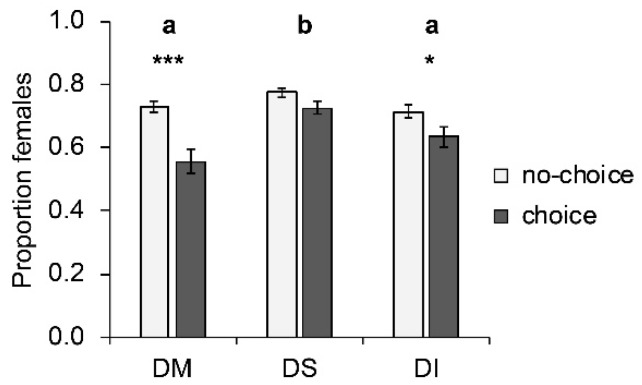
Mean (± SE) proportion of females produced by *T. drosophilae* from two strains reared for >30 generations either on *D. melanogaster* or on *D. suzukii* on different host species under no-choice (light grey) and choice conditions (dark grey). DM: *D. melanogaster*; DS: *D. suzukii*; DI: *D. immigrans*. Different letters above bars indicate significant differences between host species at the 5% level (GLM followed by sequential Bonferroni post hoc tests), stars indicate significant differences between choice and no-choice at the *p*-level of 0.001% (***), or 0.05 (*) (Mann–Whitney U test).

Host species significantly influenced the proportion of females among offspring (W_2, 250_ = 31.35; *p* < 0.001; Figure 3). More female offspring emerged from *D. suzukii* hosts than from *D. melanogaster* or *D. immigrans*. Whether females parasitized their hosts under choice or no-choice conditions significantly influenced the sex ratio of the offspring (W_1, 251_ = 29.40; *p* < 0.001); more females were produced under no-choice conditions. The largest change occurred on *D. melanogaster*, where the proportion of emerging females dropped significantly (*p* < 0.001) from 0.73 ± 0.02 (mean ± SE) under no-choice conditions to 0.56 ± 0.03 under choice conditions. This effect was less pronounced on *D. immigrans* (drop from 0.71 ± 0.02 to 0.63 ± 0.03; *p* = 0.041), and not significant on *D. suzukii* (from 0.77 ± 0.01 to 0.72 ± 0.02; *p* = 0.177). The interaction between choice and host species was significant (W_3 249_ = 8.25; *p* = 0.016). Parasitoid strain had no effect on the proportion of female offspring.

### 3.6. Behavioral Assay on First Host Choice

**Figure 4:**
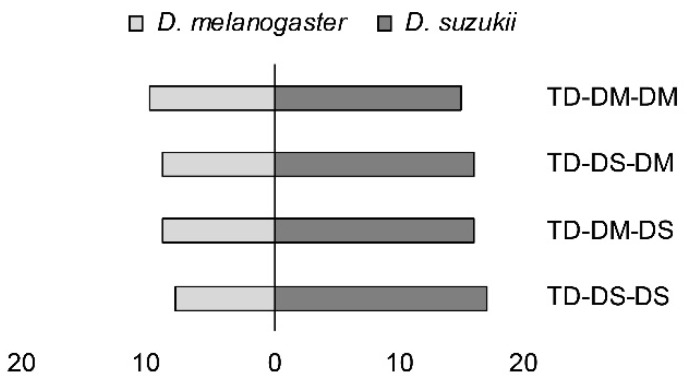
First host choice, measured as stinging into a host pupa by females from two strains of *Trichopria drosophilae* that were reared for >30 generations either on *D. melanogaster* or on *D. suzukii*. *T. drosophilae* parasitoids (TD) were reared on either *D. melanogaster* (DM) or *D. suzukii* (DS), and were then allowed one passage through either host (DM or DS) in the generation prior to the experiment.

Loglinear analysis demonstrated a significant preference for *D. suzukii* pupae by all four experimental parasitoid groups: TD-DM-DM, TD-DM-DS, TD-DS-DM, and TD-DS-DS (X^2^ = 7.95; *p* = 0.005) (Figure 4). No higher order interactions were found to have a significant effect.

## 4. Discussion

In our study, we have assessed the suitability of *D. suzukii* as a host for *T. drosophilae* in comparison to other native *Drosophila* species, and whether the rearing host affected both host preference and suitability.

Under no-choice conditions, a similar number of *T. drosophilae* offspring emerged from *D. melanogaster* and *D. suzukii*, whereas about 25% fewer individuals were produced on *D. immigrans*. It is highly unlikely that the difference in *T. drosophilae* performance was caused by differences in the health status of the hosts, since the number of emerging flies from unparasitized pupae did not differ between *D. suzukii* and *D. immigrans*. Two factors might have contributed to the observed lower number of parasitoid offspring from *D. immigrans*. First, the number of eggs laid might have been lower on *D. immigrans*, for example, due to longer handling time of the larger pupae, caused by the thicker wall of the puparium [40]. Evidence for this comes from the facts that the proportion of un-parasitized host pupae (visible as the number of emerged flies in tubes that contained a parasitoid) was higher in *D. immigrans* (17%) than in *D. suzukii* (12%). Second, the mortality of the developing parasitoids might have been higher in *D. immigrans*, as indicated by the fact that the number of pupae from which neither a fly nor a parasitoid emerged was higher in tubes with a parasitoid (25%) than in the respective control (16%). No such difference was observed for the two other hosts (Table S1). Parasitoid offspring that emerged from *D. immigrans* were heaviest, followed by individuals developing in *D. suzukii* and *D. melanogaster*, reflecting the pupal size of the *Drosophila* species. Size in parasitoids is constrained by host size, as the host presents the only available food source during development [39,41]. Larger size in insects is generally linked to greater longevity and higher fecundity [36,42]. Thus, larger parasitoids resulting from larger hosts should have a higher fitness than smaller ones. This has been observed, for example, in *Dirhinus giffardii* Silvestri (Hymenoptera: Chalcididae) parasitizing fruit fly species of different size [43]. Accordingly, we found that *T. drosophilae* emerging from *D. suzukii* produced more offspring than the smaller individuals that emerged from *D. melanogaster*. Interestingly, in the largest host, mortality was higher than in the two smaller ones. Therefore, in this host, which is phylogenetically more distant from *D. melanogaster* and *D. suzukii* [44], other factors than size, such as nutritional value, physical quality, or defense, seem to influence host quality. While we did not test whether the larger size of offspring from *D. immigrans* would translate into higher fitness of the surviving individuals, *T. drosophilae* that were reared on *D. hydei* Sturtevant, a host of similar size to *D. immigrans*, were not only larger, but also lived longer and produced more offspring [45].

When local adaptation occurs, this would become obvious in the outcome a reciprocal transplant experiment [46]. In our experiments, however, no interaction between the *T. drosophilae* strain and host species became visible in the data for offspring number or weight. Thus, the parasitoids did not perform better on the familiar host compared to the non-familiar one. Even though parasitoids had been reared on their particular host species for more than 30 generations, they were immediately able to adapt to the size of a non-familiar host by phenotypic plasticity, i.e., the capacity of a genotype to express different phenotypes according to the environment [47]. 

When given the choice between all three *Drosophila* hosts, *T. drosophilae* produced more offspring on *D. suzukii* than on the other two hosts. Although they used a much smaller number of hosts in their choice experiment, Wang et al. (2016) [23] also found a preference of *T. drosophilae* for *D. suzukii* over *D. melanogaster*; the *T. drosophilae* in their experiment, however, were all reared on *D. suzukii*. We furthermore observed that more female offspring emerged from *D. suzukii* pupae than from *D. melanogaster* or *D. immigrans* in a choice situation. This is in contrast to the results from the no-choice experiments, where similar sex ratios on the three hosts occurred. Therefore, the observed differences did not result from differential mortality, but from differences in egg laying decisions by the females. Parasitoids are able to assess host quality via visual, tactile, and chemical cues on the host surface [48]. Furthermore, they are able to adjust their sex ratio according to the perceived host quality by selectively fertilizing eggs [38]. Because female fitness is directly linked to size, higher quality hosts are used for female offspring [49]. Our results demonstrate that *D. suzukii* has been perceived as the highest quality host among the three *Drosophila* species offered. While *D. melanogaster* pupae are lighter than *D. suzukii* pupae and thus probably provide less resources, resulting in smaller and less fit offspring, *D. immigrans* are heavier and visibly larger than the two other hosts. However, wasps seem not to perceive larger hosts as high-quality hosts per se.

Even though *D. suzukii* was always preferred in the choice assay, the preference of females from the TD-DS strain was significantly larger than preference of females from the TD-DM strain. This shows that the natal host influenced host choice, even though this effect was overlain by the preference for *D. suzukii*. Preimaginal conditioning [3], as well as the exposure to host related cues during an early adult life-stage [50] can influence parasitoid host searching and preference (phenotypic plasticity). In another *Trichopria* species, *T. nigra*, Ferrero [51] found that the natal host caused a switch in preference between the two fly species *Musca domestica* and *Stomoxys calcitrans* (both: Diptera: Muscidae). Likewise, in *Aphidius ervi* (Hymenoptera: Braconidae), host fidelity occurred after one generation due to phenotypic plasticity [2]. Females of the TD-DS strain in our experiment had been continuously reared on *D. suzukii* over many generations, including the generation that was tested in the assay. Therefore, it is not possible to disentangle any genetic component in the modulation of host preference from phenotypic plasticity. In the behavioral experiment, all four tested *T. drosophilae* groups with different rearing histories showed a similar preference for *D. suzukii*, irrespective of their genetic background or the host they had emerged from. It might be that our assay was not suitable to detect subtle effects caused by genetic adaptation or phenotypic plasticity. It is also possible that associative learning after the first oviposition experience reinforces response to olfactory cues, and thus leads to stronger preferences in subsequent ovipositions [52].

In conclusion, no strong signs for artificial evolution after multiple years of rearing in the laboratory could be found in *T. drosophilae*. In general, populations develop local adaptation when selection pressures differ between environments [53]. In insects, adaptation to food sources can occur rapidly, within few generations [54,55,56]. In parasitoids in particular, the potential for divergent selection has been proposed, due to the close association with their host [57,58]. However, rapid host adaptation in parasitoids seems to be mainly present when hosts are strongly defended, whereas otherwise, little or no selection pressure is imposed by a host switch [6]. For example, two aphid parasitoid species rapidly adapted towards hosts that were infected with the protective endosymbiont *Hamiltonella defensa* (Enterobacteriales: Enterobacteriaceae) [59,60]. Likewise, in *Venturia canescens* (Hymenoptera: Ichneumonidae), a koinobiont parasitoid of pyralid moths, host switching between *Plodia interpunctella* and *Ephestia kuehniella* (both: Lepidoptera: Pyralidae) was accompanied by initial fitness costs that decreased over the course of just three generations [5]. In this case, both hosts could respond to the parasitoid egg with an encapsulation response. While in Drosophila larvae, in particular *D. suzukii*, encapsulation is known as a defense against parasitoid eggs [61], to our knowledge, no such immune response is known from *Drosophila* pupae. Moreover, phenotypic plasticity can be favored in unpredictable environments, as it allows for rapid adaptation when necessary [46,62]. Zepeda-Paulo et al. [62] observed that several populations of *A. ervi* that were collected from different hosts in the field accepted their natal host faster than non-natal hosts, while fitness did not show local host adaptation. Aphid populations in the field are highly variable in space and time, and thus comprise an unreliable food source. *Trichopria drosophilae* parasitizes a range of frugivorous *Drosophila* species developing on decaying fruit. This resource is highly variable, in particular in natural habitats, where wildfruits ripen in different places at different times and become colonized by different *Drosophila* species [63]. It is therefore possible that a high degree of phenotypic plasticity is advantageous for *T. drosophilae* under natural conditions.

Finally, genetic adaptation requires a certain amount of genetic variability as a prerequisite [46]. Our culture was founded on about 50 individuals collected in a relatively small area with *D. melanogaster* as bait [31]. It is possible that this and the subsequent laboratory culturing decreased the genetic makeup of the population in a way that affected host adaptation. 

## 5. Conclusions

*Trichopria drosophilae* is currently considered a biological control agent against *D. suzukii* in invaded areas. Therefore, a suitable rearing host, high efficacy on the target pest and limited effects on non-target species would be desirable. Our study showed that rearing *T. drosophilae* on *D. melanogaster* for more than 30 generations does not influence its performance on the target *D. suzukii*, and therefore stock rearing can be performed on this easy to handle host. However, offspring that emerged from *D. suzukii* had a higher egg laying capacity and an increased preference towards the host. It may therefore be advisable to pass *T. drosophilae* through *D. suzukii* prior to release when using another *Drosophila* species for mass rearing. Furthermore, the seemingly innate preference for *D. suzukii* over *D. melanogaster* and *D. immigrans* holds promise that this species would also be attacked preferentially in the field. However, one has to consider that there are many more species of *Drosophila* present in the field that have not been tested in this study.

## Supplementary Materials

The following are available online at https://www.mdpi.com/2075-4450/10/6/183/s1, Table S1: Detailed experimental data.
